# Exploitation of SPR to Investigate the Importance of Glycan Chains in the Interaction between Lactoferrin and Bacteria

**DOI:** 10.3390/s17071515

**Published:** 2017-06-27

**Authors:** Noelle O’Riordan, Michelle Kilcoyne, Lokesh Joshi, Rita M. Hickey

**Affiliations:** 1 Biosciences Department, Teagasc Food Research Centre, Moorepark, Fermoy, P61C996 Co. Cork, Ireland; noriordan1@gmail.com; 2 Glycoscience Group, National Centre for Biomedical Engineering Science, National University of Ireland Galway, H91TK33, Galway, Ireland; michelle.kilcoyne@nuigalway.ie (M.K.); lokesh.joshi@nuigalway.ie (L.J.)

**Keywords:** lactoferrin, glycosylation, surface plasmon resonance, bacterial binding, lactation

## Abstract

Bovine lactoferrin (LF) has been shown to prevent adhesion to and invasion of mammalian cell lines by pathogenic bacteria, with evidence for direct bacterial binding by the milk glycoprotein. However, the glycosylation pattern of LF changes over the lactation cycle. In this study, we aim to investigate the effect that this variation has on the milk glycoprotein’s ability to interact with pathogens. Surface plasmon resonance technology was employed to compare the binding of LF from colostrum (early lactation) and mature milk (late lactation) to a panel of pathogenic bacteria (*Staphylococcus aureus*, *Escherichia coli*, *Cronobacter sakazakii*, *Streptococcus pneumoniae*, *Pseudomonas aeruginosa*, *Listeria monocytogenes* and *Salmonella typhimurium*). Novel interactions with LF were identified for *C. sakazakii*, *S*. *pneumoniae* and *P*. *aeruginosa* with the highest binding ability observed for mature milk LF in all cases, with the exception of *S*. *typhimurium*. The difference in bacterial binding observed may be as a result of the varying glycosylation profiles. This work demonstrates the potential of LF as a functional food ingredient to prevent bacterial infection.

## 1. Introduction

Bovine milk lactoferrin (LF) is a single chain, iron-binding, glycosylated protein [1] present in the whey protein fraction of milk [2] (http://www.uniprot.org/uniprot/B9VPZ5). There are two lactoferrin variants present in bovine milk: LF-a and LF-b. Five glycosylation potential sites (Asn^233^, Asn^281^, Asn^368^, Asn^476^ and Asn^545^) were identified through a combination of digestion and HPLC on LF, of which four are always utilised but Asn^281^ is glycosylated only on LF-a [3,4,5]. Lactoferrin may have high-mannose type, complex type or hybrid type N-linked glycans attached. The associated glycan chains are composed of N-acetyl-glucosamine (GlcNAc), galactose (Gal), N-acetyl-galactosamine (GalNAc), fucose (Fuc), mannose (Man), N-acetyl-neuraminic acid (Neu5Ac) and N-glycolyl-neuraminic acid (Neu5Gc) (as previously reviewed [6]) and variation in the glycosylation pattern of LF has been described over the lactation cycle [7]. A greater degree of heterogeneity in glycan structure has been reported for LF from early lactation, with an abundance of high-mannose type glycans present on th glycoprotein in mature milk [7]. The glycan chains in mature milk LF are 65% oligomannose type, consisting of multiple isomers of high-mannose type glycans, containing five to nine mannose residues, 38% of the total mannose structures accounted for by Man8 [8]. Neu5Gc was shown to be present on bovine LF only in the initial days postpartum and the total sialic acid content decreased as lactation progressed [7].

LF has a wide variety of associated biological activities including antimicrobial [9], immunomodulatory [10], prebiotic [11,12], stimulation of bone formation [13] and anticancer [14,15]. These bioactivities have previously been reviewed by several authors [16,17] and the glycan component of LF has an important role to play in many of these properties [6]. The presence of the glycan chains influences the tertiary structure of the protein, and enhances its ion binding ability [18,19,20] and its resistance to enzyme digestion [21]. The attached glycans are also believed to have a role in LF’s antibacterial [22,23,24] and antiviral [25,26] activities. However, to date, information on how glycosylation variation over lactation influences bioactivity is limited.

Biomolecular interaction analysis (Biacore) involves the use of surface plasmon resonance (SPR) to measure the binding of candidate compounds or cells to specific ligands. SPR measures the changes in the refractive index near a planar chip surface induced by binding of soluble molecules to immobilized counterpart molecules on the sensor chip [27]. It has been widely used for the quantification and kinetic analysis of receptor-ligand interactions [28,29,30,31,32,33]. Recently, this methodology has been identified as a reliable, high throughput method for profiling the interaction of whole bacterial cells with an immobilised glycans [28]. The authors validated the Biacore assay by investigating the interaction between *Campylobacter jejuni* and the milk oligosaccharide, 2’-fucosyllactose, to which it is known to bind [34,35]. The assay monitors interactions dynamically over time in a continuous flow system and may mimic natural conditions in a more realistic manner when compared with static adhesion assays [28]. A number of similar studies to monitor whole bacterial cell interactions with immobilised compounds of interest have also been documented [36,37,38,39]. Of particular interest is a study using SPR to distinguish between five closely related *Escherichia coli* strains, including the enterohemorragic *E. coli* O157:H7 based on direct differential carbohydrate recognitions [40].

The current study aims to exploit SPR to investigate the importance of the glycan chains in the interaction between LF and bacteria. Commercially available LF from colostrum and mature bovine milk was biotinylated and immobilised on the streptavidin coated surface of a Streptavidin SA chip. A number of pathogenic bacterial strains were selected and screened for interactions with both glycovariants.

## 2. Materials and Methods

### 2.1. Materials

LF from colostrum and mature milk was purchased from Sigma-Aldrich Co. (Dublin, Ireland). Mueller-Hinton broth and Brain Heart Infusion broth were purchased from Oxoid (Basingstoke, Hampshire, UK). The Biacore X instrument, SA chip, biotin CAPture kit, HBS-EP buffer (10 mmol·L^−1^ Hepes, 150 mmol·L^−1^ NaCl, 3.8 mmol·L^−1^ ethylenediaminetetraacetic acid (EDTA), 0.05% (*v*/*v*) Tween 20), and amine coupling kit were purchased from GE Healthcare (Buckinghamshire, UK). The EZ-Link biotin-PEG4-hydrazide kit, sodium acetate buffer (pH 5.5), and Zeba desalt spin column were purchased from Pierce (Rockford, IL, USA). Sodium meta-periodate was purchased from Sigma-Aldrich Co. (Dublin, Ireland).

### 2.2. Bacterial Strains and Culture Conditions

Bacterial strains used in this study and their respective growth media are listed in Table 1.

Bacterial culture stocks were maintained in their respective growth media containing 50% glycerol at −80 °C in the culture collection at Teagasc Food Research Centre, Moorepark, Fermoy, P61C996, Co. Cork, Ireland and propagated twice prior to use. All bacterial strains were grown aerobically for 12–24 h at 37 °C. Bacterial cells were washed three times in HBS–EP buffer and re-suspended to a concentration of 1 × 10^8^ colony-forming units (CFU) mL^−1^ for screening studies unless otherwise stated.

### 2.3. Biotinylation of Lactoferrin

LF samples were biotinylated using EZLink biotin-PEG4-hydrazide as per the manufacturer’s instructions. Briefly, 2 mg of LF was dissolved in 1 mL of 0.1 mol·L^−1^ sodium acetate buffer (pH 5.5). An amount of 1 mL of cold sodium meta-periodate solution (20 mmol·L^−1^ periodate) was added and the solution was mixed well. The mixture was protected from light and incubated for 30 min at room temperature. Excess periodate was removed using a Zeba desalt spin column equilibrated with 0.1 mol·L^−1^ sodium acetate buffer (pH 5.5). One part of 50 mmol L^−1^ biotin-hydrazide solution was added to nine parts of the treated sample and incubated for 3–4 h at room temperature. Unbound biotin remaining in the sample was removed by passing the solution sequentially through two Zeba desalt spin columns. The sample was then stored at 4 °C until use.

### 2.4. Biacore Assay

The entire analysis was carried out on a Biacore X instrument at a constant temperature (25 °C) and flow rate (10 µL·min^−1^), unless otherwise stated, using HBS–EP as the run buffer and a SA chip. The streptavidin-coated SA chip surface was primed with a short injection of 1 mol·L^−1^ NaCl and 50 mmol·L^−1^ NaOH (filtered and degassed). Whole bacterial cells were harvested and resuspended in HBS–EP running buffer as described in Section 2.2. In order to confirm the absence of non-specific binding of the selected bacterial strains to the SA chip surface, cell suspensions (1 × 10^8^ CFU mL^−1^) were injected over the chip surface at 10·µL·min^−1^, and the binding signal was measured. Signal change was reported in response units (RU). The chip surface was washed with HBS–EP running buffer between bacterial injections to ensure full removal of microbial cells.

Thereafter, biotin-labelled LF (from either colostrum or mature milk) was diluted in HBS–EP buffer (50 µg·mL^−1^), and 100 µL of this solution was injected over the surface at a flow rate of 10 µL·min^−1^. The chip surface was then washed with HBS–EP buffer to ensure the removal of any non-immobilized molecules. The RU increase following each LF injection was monitored to ensure comparable levels of analyte were immobilised. Subsequently, bacterial injections were repeated to evaluate bacterial binding to immobilised colostrum and mature milk LF.

### 2.5. Statistical Analysis

All experiments were performed in triplicate and results are presented as mean values ± standard deviations of three replicate experiments. Nonspecific binding of the analyte to the test surface was eliminated from all experiments through the use of a reference surface.

## 3. Results

In the current study, SPR was used to investigate the effect of changes in LF glycosylation over lactation on its ability to bind to pathogenic bacteria. A panel of pathogenic bacteria was initially exposed to the surface of the SA chip, minus the immobilised analyte, to ensure the absence of non-specific binding to the chip surface, which could result in false results during the test runs. Bacterial solutions were washed and resuspended in buffer prior to exposure to the chip surface to ensure no salts were present in the samples. The chip was also washed between exposures to different bacterial strains and also on a daily basis. The system was also optimized in terms of the flow rate to ensure that cell deposition did not occur. For all bacteria screened, non-specific binding was not observed (data not shown). Concentrations of bacteria at approximately 1 × 10^8^ CFU·mL^−1^ were used in the assays as previous studies have shown that concentrations in this range are generally required to generate a measurable signal [28].

**Figure 1:**
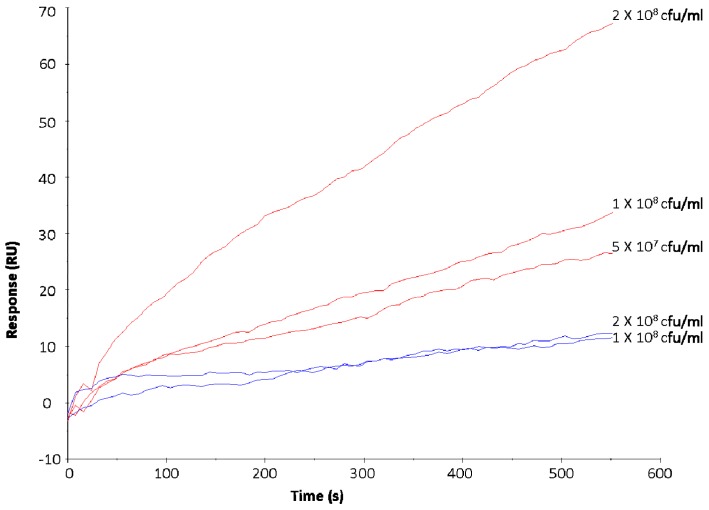
Increasing concentrations of *Staphylococcus aureus* DPC 5971 (red) and *Escherichia coli* O157:H7 P1432 (blue) injected over the surface of a streptavidin (SA) chip with mature LF immobilised to evaluate effect of increasing bacterial numbers on the response units (RU) response.

Based on previous studies [23,41,42] and the results generated in the initial phase of this work, *S. aureus* DPC 5971 and *E. coli* P1432 were identified as positive and negative controls for binding to LF respectively. Exposure of bacterial suspensions with increasing cell number resulted in an increase in the RU value with *S. aureus* DPC 5971, confirming that the injected bacterial cells were interacting with the immobilised LF (Figure 1). It should be noted that at the end of the injection period of 600 s, the RU values were maintained before a gradual decline in RU was observed, indicating the specific nature of the interactions (data not shown).

No increase in RU value was observed for *E. coli* P1432 following exposure of increasing concentrations of bacterial cells to immobilised LF (Figure 1), confirming the lack of binding of this strain to the analyte. These strains were exposed to the surface of the SA chip with immobilised LF at the beginning of each experimental set to ensure consistent performance of the chip. The response of *E. coli* P1432 (colostrum: 16.8 ± 4.3 RU; mature milk: 17 ± 7.1) was considered a baseline minimum RU value and used as a reference for screening the other pathogens for positive interactions.

**Figure 2:**
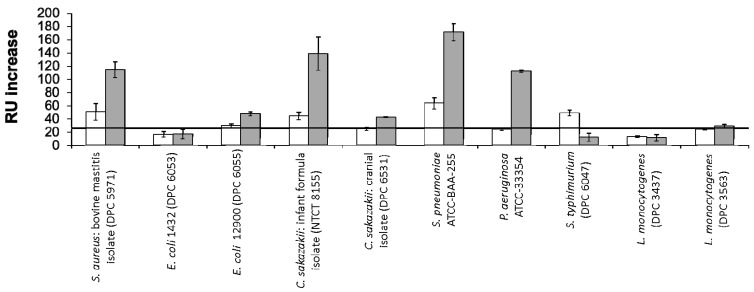
RU changes following exposure of a panel of pathogenic bacteria to LF from colostrum (white) or mature milk (grey) immobilised on a Biacore SA chip. *E. coli* P1432 was identified as a negative control and its response was marked as the minimum requirement for consideration as a positive interaction (black horizontal line). All bacteria were applied at a concentration of 1 × 10^8^ CFU·mL^−1^.

RU changes following exposure of the bacterial strains to the immobilised colostrum and mature milk LF are shown in Figure 2. Of the positive interactions profiled, the strongest signals were observed for *C. sakazakii* NCTC 8155, *S. pneumoniae* ATCC BAA-255 and *P. aeruginosa* ATCC 33354. Neither *L. monocytogenes* strain tested displayed binding to either LF glycovariant. For the majority of strains which displayed positive binding, a stronger interaction was observed for mature milk LF versus colostrum LF. *S. typhimurium* ATCC BAA-185 was the sole exception to this, interacting only with the glycovariant of the protein isolated from colostrum.

## 4. Discussion

The greater interaction with mature LF may be linked to the abundance of high-mannose type glycans on this glycovariant. Teraguchi, Shin, Fukuwatari and Shimamura [23] described the relationship between LF and members of the *Escherichia* family and the dependence of the observed interaction on the presence of high-mannose type glycans on the milk protein. Members of this family which express type 1 fimbriae can recognise and bind to oligomannose glycan chains on eukaryotic cell surfaces [43], which can facilitate bacterial adhesion to and invasion of the cells. Teraguchi et al. [23] demonstrated that the high mannose-type glycans on bovine LF acted as receptors for the mannose specific type 1 fimbriae, therefore preventing bacterial interaction with the eukaryotic cell by acting as a decoy receptor. When human LF was tested, this activity was not observed, potentially as a result of the presence of only complex type glycans on the human protein variant. The same group went on to demonstrate that bovine LF caused the agglutination of type 1 fimbriated *E. coli* cells as a result of the specific interaction between the mannose residues on the glycoprotein and the type 1 fimbriae of the bacteria. The results presented here suggest that bovine LF may also have similar interactions with other pathogenic bacteria as a result of its direct bacterial interaction via its oligomannose type glycans. The glycoprotein may have a role as a more non-specific defence mechanism, inhibiting bacterial adhesion to mammalian cells.

The lower binding observed for LF isolated from early lactation may also be linked to the presence of more diverse antimicrobial elements in colostrum such as immunoglobulins and oligosaccharides. Therefore, there may be less of a biological requirement for LF to contribute to the inhibition of pathogenic infection. Also, the presence of sialic acid on colostrum LF glycans suggests that this glycovariant may have an alternative method of anti-bacterial activity in early lactation. Sialylation has previously been linked to LF’s antimicrobial activity as a result of its calcium chelation activity, competing for loosely bound calcium ions involved in the stabilization of lipopolysaccharides in the outer membrane of bacterial cells [18,44].

*S. aureus* DPC 5971 is a bovine mastitis isolate [45] and was selected as the positive control for this study as receptors for LF have previously been identified in *S. aureus* strains associated with mastitis infection [41]. The LF concentration in milk has been shown to increase in cows suffering from subclinical mastitis [46], possibly as part of an immune response to infection. The results of this study demonstrate that although *S. aureus* DPC 5971 binds to both colostrum and mature milk LF, the glycoprofile present on mature milk LF is more favourable for bacterial binding, suggesting that the *S. aureus* receptors which interact with LF are regulated by the glycosylation present on the protein.

Two *E. coli* O157:H7 strains were included in this study: *E. coli* P1432 and *E. coli* NCTC 12900; the former was included as the negative control. O157 strains have previously been shown to be unable to produce type 1 fimbriae (because of a deletion in the *fim* regulatory region) [42] described by Teraguchi, Shin, Fukuwatari and Shimamura [23] as the mode of binding for *E. coli* cells to LF mannose residues. Of interest, a recent study proposes a method for pathotyping *E. coli* based on type 1 fimbrial interaction with a mannosylated surface using shear force [47]. Minimal binding was observed for *E. coli* NCTC 12900 with mature milk LF. LF has been shown to bind to different *E. coli* strains with varying levels of efficiency, for both the human and bovine variants of the protein [48,49].

Two *C. sakazakii* strains were exposed to immobilised LF in this study: *C. sakazakii* NCTC 8155, a strain isolated from dried milk powder; and *C. sakazakii* DPC 6531, a brain tumour isolate. The most significant binding was observed for *C. sakazakii* NCTC 8155. LF has been shown to display anti-bacterial [50,51] and anti-infective [52] activity against *C. sakazakii*. This study confirms the direct binding of LF to *C. sakazakii*, which may give further insight into its mode of antibacterial action, which to date is believed to be dependent on the protein’s iron chelating ability. Galactoligosaccharides [53] and LF-containing bovine whey powders [54] have previously been shown to inhibit the adherence of *Cronobacter* strains to gastrointestinal cells and in some cases prevent invasion. For both strains, a higher RU value was noted when the immobilised analyte was the mature milk glycovariant, again highlighting the importance of the mature milk glycoprofile in bacterial binding. In relation to the strain specificity observed for *C. sakazakii*, LF binds more intensely to the strain isolated from milk powder. Milk powder is a common source of *C. sakazakii* contamination and the World Health Organisation (WHO) has issued guidelines relating to the recommended minimum temperature for reconstitution to reduce infection risk. Fortification of milk powders and infant milk formulas with LF as a secondary hurdle to infection may further reduce the annual incidences of *C. sakazakii* infection, which are most common in neonates, infants and immunocompromised adults.

Novel interactions for *S. pneumoniae* ATCC BAA-255 and *P. aeruginosa* ATCC 33354 with LF were observed in this study, both interacting to a greater extent with the glycoprotein from mature milk. Two proteins from *S. pneumoniae* which bind to human LF have previously been identified [55]. These proteins have been suggested to be elements of a novel virulence mechanism, harnessing LF’s iron binding activity to overcome iron limitation at mucosal surfaces. Antibacterial activity against *P. aeruginosa* has been observed upon exposure to a peptide derived from LF, lactoferricin B [56]. However, this is the first study, to the best of our knowledge, that demonstrates binding of bovine milk derived LF to the whole bacterial cells. Both *S. pneumoniae* [57] and *P. aeruginosa* [58] have previously been shown to have affinity for neutral oligosaccharides with terminal D-Gal residues, suggesting that the complex type glycans in mature milk have a role to play in the binding of these strains. Complex glycans have a higher level of sialylation in colostrum [7], inhibiting recognition of terminal Gal residues by bacterial lectins. Therefore, the mature milk glycoprofile offers terminal D-Gal residues as targets for bacterial binding.

*S. typhimurium* was the sole bacterial strain to show greater binding to colostrum LF. *S. typhimurium* adhesion to intestinal cells has previously been shown to be linked to cellular glycosylation and can be inhibited by lectins PNA, AIA, ECA, RCA I and WGA which compete for the glycan receptors including Galβ(1–3)GalNAc on the epithelial cell surface [59]. Binding patterns of these lectins to LF over lactation was previously profiled and all lectins displayed the highest interaction with colostrum LF [7], suggesting the glycosylation profile of LF from early lactation is in some way homologous to the binding sites on Caco-2 cells for *S. typhimurium*. Although cows of all ages can be infected with Salmonella bacteria, serious infections and deaths are most often seen in young calves [60]. The glycosylation pattern of LF in early lactation may contribute to the immune protection delivered to the calf via its mother’s milk and may have evolved to offer protection against infections most likely to occur in early life.

Colostrum LF has a higher N-acetylneuraminic acid (Neu5Ac) content compared to mature milk LF [7] and this monosaccharide is closely linked to LF’s antibacterial activity, as previously reviewed [6]. However, minimal bacterial binding to colostrum LF was observed in this study. This suggests that bovine LF has two modes of action for its anti-microbial activity; transitioning from a Neu5Ac dependent functionality in early lactation, to a more non-specific role in mature milk as a decoy receptor, which can bind a number of pathogenic bacteria, preventing adhesion and infection. This would suggest LF’s glycosylation changes over the lactation period to deliver the most optimum protection relevant to the life-stage of the offspring.

## Figures and Tables

**Table 1:** List of bacterial strains tested.

Strain	Media
*Staphylococcus aureus* DPC 5971	Muller-Hinton
*Escherichia coli* O157:H7 P1432	Muller-Hinton
*Escherichia coli* O157:H7 NCTC 12900	Muller-Hinton
*Cronobacter sakazakii* NCTC 8155	Brain Heart Infusion
*Cronobacter sakazakii* DPC 6531	Brain Heart Infusion
*Streptococcus pneumoniae ATCC BAA*-*255*	Todd Hewitt + 0.5% yeast extract
*Pseudomonas aeruginosa ATCC 33354*	Tryptic soy broth
*Salmonella enterica* subsp. enterica serovar Typhimurium *ATCC BAA*-*185*	Brain Heart Infusion
*Listeria* *monocytogenes DPC 3437*	Brain Heart Infusion
*Listeria* *monocytogenes* NCTC 11994	Brain Heart Infusion
